# Decompressive Hemicraniectomy for a Patient With Intraventricular Hemorrhage Due to a Ruptured Arteriovenous Fistula and Refractory Intracranial Hypertension

**DOI:** 10.7759/cureus.97952

**Published:** 2025-11-27

**Authors:** Jessica Sawaya, Rasha Elbadry, Promod Pillai, Tanya Minasian

**Affiliations:** 1 Department of Neurological Surgery, Loma Linda University School of Medicine, Loma Linda, USA; 2 Department of Neurological Surgery, Loma Linda Medical Center, Loma Linda, USA

**Keywords:** artiovenous fistula, decompressive craniectomy, hydrocephalus, intracranial hemorrhage, intracranial hypertension

## Abstract

Arteriovenous fistulas (AVFs) are a rare disease process occurring in pediatric patients with presentations ranging from asymptomatic to intracranial hemorrhage. Intracranial hypertension secondary to AVF rupture increases the risk of further neurological sequelae and hemodynamic instability, requiring immediate stabilization in the acute setting. Traditional treatments for AVF include endovascular embolization, microsurgical disconnection, or a hybrid approach; however, increased intracranial pressures secondary to intracranial hemorrhage or hydrocephalus may necessitate other forms of acute intervention such as external ventriculostomy drains (EVDs), hemorrhagic evacuation, or decompressive craniectomy. Here, we present a five-year-old, previously healthy, female who presented with a tonic-clonic seizure following two weeks of headache, nausea, and emesis after a fall. Imaging demonstrated intraventricular hemorrhage throughout the entire ventricular system secondary to a ruptured pial AVF (pAVF). Due to significantly increased intracranial pressure refractory to medical management, decompressive craniectomy was conducted, followed by endovascular embolization of the arteriovenous nidus. The patient made an excellent neurological recovery without recurrence of bleeding or residual neurological deficits. This represents the successful management of refractory intracranial pressure due to intraventricular hemorrhage and arteriovenous fistula using decompressive craniectomy despite the absence of cerebral edema or lateralizing pathology, leading to an excellent patient recovery.

## Introduction

Pial arteriovenous fistulas (pAVFs) are rare vascular lesions in which one or more arterial vessels drain directly into a venous channel [1]. They occur most commonly in the pediatric population but still represent a very small proportion of intracranial vascular malformations, with an incidence ranging from 1.6% to 7.3% [1-4]. pAVFs differ from arteriovenous malformations such that pAVFs lack an intervening nidus between the feeding artery and draining vein, creating a higher velocity of flow and making the lesion more vulnerable to rupture and resulting in intracerebral hemorrhage [1, 4]. pAVFs can be acquired or congenital in etiology; however, they are more commonly congenital and may be associated with certain syndromes, including Rendu-Osler-Weber disease, Klippel-Trenaunay-Weber syndrome, Ehlers-Danlos syndrome, and hereditary hemorrhagic telangiectasia [1,2,5]. Clinical presentation varies from asymptomatic to manifestations of intracranial hypertension or intracerebral hemorrhage, including headache, seizures, and neurological deficits [1].

If left untreated, pAVF has a mortality rate of 63% [2]. As a result of this high risk of mortality and neurocognitive prognosis, occlusion of the arteriovenous shunt is necessary [6]. The primary goal of treatment is disconnection of the pAVF shunt without necessarily resecting the vascular malformation [1, 2, 6]. This can be accomplished through an endovascular or surgical approach, although the endovascular approach is demonstrated to be the preferred method due to its noninvasive and safer nature [1, 4]. These treatment options may be complicated by pAVF in a deep-seated location, surrounding critical structures, with a short arterial feeder, or in the case of rupture, refractory to medical management [1]. While the standard approach for intracerebral hemorrhage secondary to other vascular pathologies, such as arteriovenous malformations, cavernous malformations, and cerebral aneurysm ruptures, includes surgical resection of the lesion or endovascular therapy, the management and timing of pAVF rupture are still unclear [7, 8]. Here, we present the case of a patient with complicated pAVF rupture to illustrate the role of decompressive craniectomy as a lifesaving intervention in refractory intracranial hypertension due to this pathology.

## Case presentation

**Figure 1:**
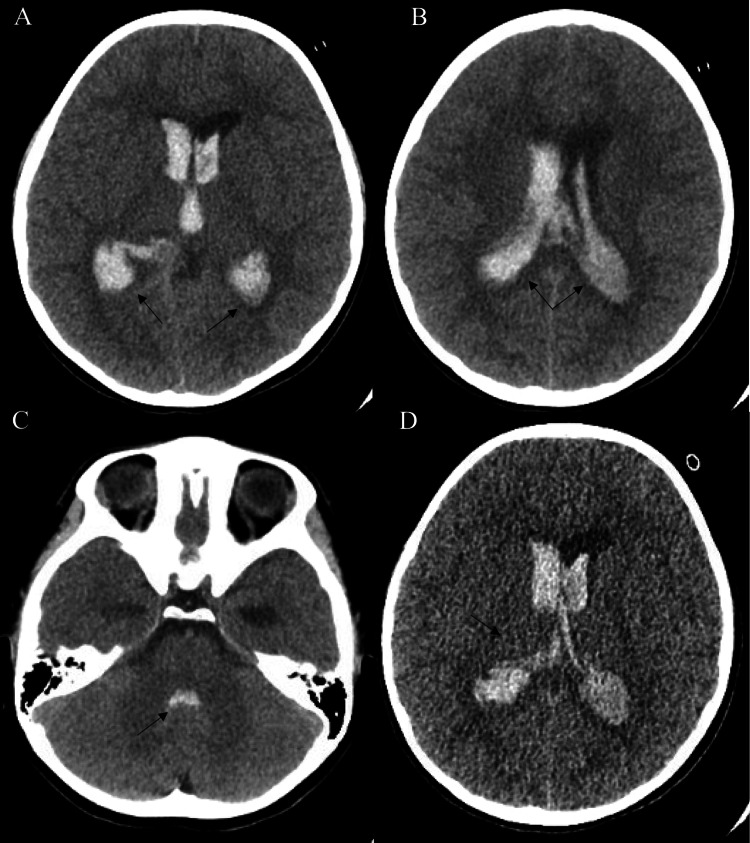
Computed tomography (CT) head without contrast (A) Extensive hemorrhage in the ventricular system with interval dilatation of the temporal horns (black arrows) of both right and left lateral ventricles. (B) Extensive hemorrhage in the lateral ventricles (black arrows). (C) Hemorrhage in the fourth ventricle (black arrow). (D) Hemorrhage likely originating from the right thalamus (black arrow).

We present a five-year-old previously healthy female patient who developed continuous headaches, nausea, and nonbilious nonbloody emesis for approximately two weeks after falling off a swing. The patient presented to an outside emergency department after a tonic-clonic seizure with a Glasgow Coma Score (GCS) of three and was subsequently intubated for airway protection. An outside hospital computed tomography (CT) scan of the head revealed intraventricular hemorrhage within all four ventricles (Figure 1) originating from the region of the right thalamus, thought to be due to rupture of a vascular malformation, and a right external ventriculostomy drain (EVD) was placed. Upon arrival at our hospital, the patient was intubated with a blood pressure of 118/90 mmHg, a pulse of 128 beats per minute, respirations of 30 breaths per minute, and a temperature of 97.6 degrees Fahrenheit. The patient presented with pinpoint pupils, responsive to pain, with intact cough and gag reflexes. The right EVD had stopped draining, and intracranial pressures remained elevated, prompting left EVD placement. Despite continuous medical management, including hypertonic saline, bilateral EVD, optimized sedation, and pentobarbital bolus and drip, the patient’s intracranial hypertension remained refractory, with intracranial pressures spiking as high as 50 mmHg. It was decided to proceed with a right hemicraniectomy for decompression of the brain with a left intracranial pressure monitor/bolt placement on hospital day two. 

A right frontal temporal parietal reverse question mark incision was made with a #15 blade and was extended through the galea, temporalis muscle, and fascia with monopolar cautery. The myocutaneous scalp flap was elevated together, dissected from the skull, and retracted anteriorly with fish hooks. Three burr holes were made with the perforator, one above the root of the zygoma and the other two anterior and posterior off the midline. The dura was dissected from the inner table of bone with a Penfield, and a craniotome was used to create a large craniotomy. The bone in the middle fossa was removed with a rongeur to allow for extensive temporal lobe decompression. The dura was noted to be bulging and excised with a #15 blade and opened with tenotomy scissors. There was significant edema out of the craniotomy defect, engorged cortical veins, and subarachnoid hemorrhage noted throughout. On a separate incision in the left frontal region, the intracranial pressure anchor bolt was secured in place. 

**Figure 2:**
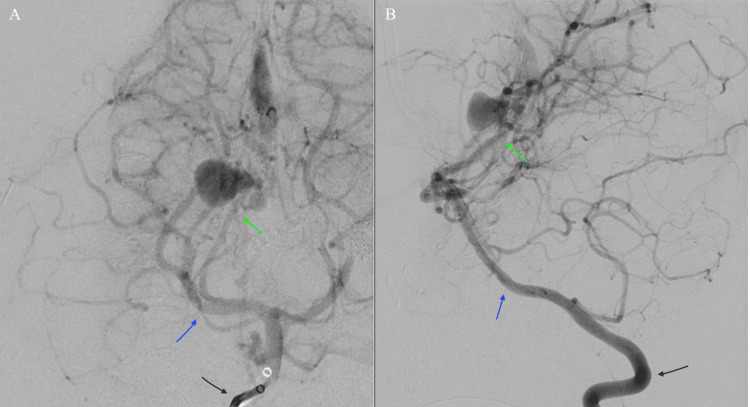
Digital subtraction angiography (A) Coronal and (B) lateral views showing brisk anterograde opacification of the right vertebral artery (black arrow) and its major branches with arterial phase opacification of the basal vein of Rosenthal (green arrow) via a branch of the right posterior cerebral artery (blue arrow) near the pial arteriovenous fistula.

Postoperatively, the patient remained intubated on sedation with hypertonic saline and a norepinephrine drip to maintain cerebral perfusion, with intracranial pressure measuring at 16 mmHg. On hospital day three, the patient underwent digital subtraction angiography and was found to have a right posterior cerebral artery basal vein of Rosenthal pAVF, which was subsequently embolized with interventional neuroradiology (Figure 2). Intracranial pressure postoperatively continued to drop, ranging between 12 and 13 mmHg. The right EVD was removed on hospital day five. The patient demonstrated a consistent reduction in intracranial pressure below 10 mmHg and was stabilized for extubation on hospital day seven. The left EVD was then removed on hospital day seventeen. The patient was discharged 13 days later on levetiracetam for seizure control and baby aspirin for post-fistula embolization. 

The patient returned approximately one month after the previous discharge for a planned right autologous cranioplasty, for which the autologous graft was secured in a bone bank, as is standard for our institution. At this time, the patient had already made an excellent neurological recovery with a return to a baseline of GCS 15 and was able to follow simple commands, speak in short word combinations, and move all extremities spontaneously. The patient was discharged on hospital day two following surgery and returned for a two-week follow-up with continued improvement in neurological status with a well-healed cranial incision without signs of infection. Two months later, the patient was walking into the clinic and was completely neurologically intact.

## Discussion

pAVF is a rare disease in pediatric patients, with one study estimating a prevalence ranging from 0.1 to one per 100,000 individuals [2]. In pAVF, a feeding artery directly communicates with a draining vein without an intervening nidus of vessels, creating a higher pressure gradient that makes the lesion more vulnerable to rupture and poor prognosis [1]. Long-term high-velocity turbulent flow further predisposes to venous varix formation, which is an additional risk factor for hemorrhage [3-5]. The likelihood of intracranial hemorrhage secondary to pAVF rupture increases with age, with a higher frequency in those over 15 years of age [2, 5]. Intracranial hemorrhage may be the initial presentation of pAVF, as seen in our patient. If symptomatic, pAVF can present with headache, seizures, focal neurological deficits, developmental delay, macrocephaly, and congestive heart failure in neonates [1, 2, 6].

The gold standard diagnosis for pAVF is digital subtraction angiography due to its superior spatial and temporal resolution to clearly delineate the fistula site, arterial supply, and venous drainage. Magnetic resonance angiography and CT angiography are other radiographic modalities to identify pAVF; however, they may be less useful for treatment planning [1, 4, 5]. Complete occlusion of pAVF can be achieved through endovascular embolization, microsurgical disconnection, or a combination of both [2, 5]. Given the generally deep-seated location and high-flow nature of pAVF, an endovascular approach is often preferred for treatment, as chosen for our patient following decompressive craniectomy [1, 5]. Surgical management is often reserved for cases where embolization is deemed too dangerous due to a short arterial feeder branching from a cortical artery that cannot be occluded [6]. 

Complications of treatment, either through the endovascular or surgical approach, include venous thrombosis, often prevented with postprocedural heparin, and intracerebral hemorrhage due to abrupt closure of a high-velocity fistula. Other complications include those associated with altered hemodynamics that interfere with normal cerebral autoregulation, resulting in cerebral edema and infarction [1]. In the pediatric population, endovascular embolization exposes patients to ionizing radiation and may fail to successfully treat high-flow lesions, leading to rupture or impaired venous outflow. On the other hand, surgical treatment increases the risk of blood loss and inability to occlude the pAVF due to deep location or impaired visualization secondary to hemorrhage [2]. Despite a complicated clinical course requiring decompressive craniectomy due to refractory intracranial hypertension, our patient demonstrated an excellent neurologic recovery without any residual deficits.

Patients with intracranial hypertension secondary to pAVF rupture are at risk of developing secondary injury, including hemodynamic instability, cardiopulmonary sequelae, and neurological deficits [7]. Other neurological complications include brain herniation, seizures, altered levels of consciousness, and eventually death if not appropriately treated in the acute phase [9]. Our patient presented with altered consciousness with a GCS of three and seizures secondary to severely increased intracranial pressure, putting her at immediate risk of significant morbidity and mortality. Patients with elevated intracranial pressure due to expansive hematoma volume or cerebral edema, as well as hydrocephalus, are more vulnerable to developing hypotension and hypoperfusion that may exacerbate ischemic injury, resulting in considerable neurological morbidity, including cognitive impairments and decreased IQ. Managing acute hydrocephalus secondary to intraventricular hemorrhage and minimizing the time with elevated intracranial pressure, therefore, serves as a key therapeutic target in the acute phase of pAVF rupture to improve neurological outcomes and reduce neurological sequelae in patients who are already at risk of neurological insults secondary to this condition [7, 10]. Decompressive craniectomy is therefore indicated in the acute setting in children with elevated intracranial pressure refractory to medical management in order to urgently control intracranial pressure and enhance neurological function and prevent secondary insults to the brain [11]. Our patient had significantly elevated intracranial pressure secondary to intraventricular hemorrhage refractory to medical management, including bilaterally placed EVDs. Given the deteriorating neurological status with which our patient presented, she underwent a subsequent right-sided hemicraniectomy to allow for decompression of the brain and more immediate control and stabilization of her intracranial pressure. Despite the absence of cerebral edema or lateralizing pathology, this approach proved effective, which allowed for excellent neurological recovery and return to baseline GCS from 3 to 15. Etter et al. reported a similar outcome in a nine-year-old child with intraparenchymal hemorrhage relieved by hematoma evacuation due to suspected pAVF rupture, suggesting that immediate surgical intervention to manage refractory intracranial pressure may be indicated to prevent serious neurological sequelae without sacrificing patient morbidity [2].

Decompressive craniectomy has been applied in the treatment of intracranial hypertension in children with various pathologies with good results [7, 12-14]. The most common causes of intracerebral hemorrhage in children include arteriovenous malformations, arteriovenous fistulas, and cavernous malformations [8]. Standard of care for arteriovenous malformation rupture and cavernous malformations is surgical resection, while standard treatment for ruptured cerebral aneurysms includes microsurgical clipping or endovascular therapy, with management of acute intracranial pressure consisting of EVDs and medical stabilization of intracranial pressure [7, 12]. In the case of refractory medical management, however, more aggressive interventions may be indicated. LoPresti et al. demonstrated that initial decompressive craniectomy for ruptured arteriovenous malformations in children did not increase the risk of morbidity and mortality compared to initial arteriovenous malformation resection and rather provided additional time for initial recovery and patient stabilization in the acute phase, which allowed for better surgical visualization for subsequent arteriovenous malformation resection [7]. Other case reports have demonstrated similar findings of good neurological recovery with decompressive craniectomy for intracranial hypertension secondary to middle cerebral artery aneurysm rupture and idiopathic thrombocytopenic purpura-related disease [12,14]. Significant benefits to decompressive craniectomy for management of intracranial pressure include rapid and persistent reduction of intracranial pressure, preservation of neurological status, and patient stabilization in the acute setting [7, 11]. In the case of concomitant intraventricular hemorrhage, as in our case, decompressive craniectomy with the addition of EVD placement for cerebrospinal fluid diversion and pAVF resection serves as an important initial treatment for large-volume hemorrhage or cerebral edema to immediately reduce the intracranial pressure [7, 8]. After acute stabilization, our patient’s pAVF was later definitively treated with endovascular embolization. This case suggests that decompressive craniectomy can be successfully applied to ruptured pAVFs in the case of elevated intracranial pressure secondary to intraventricular hemorrhage refractory to medical management, with a good outcome in neurological recovery. 

## Conclusions

A pAVF is a rare disease that may present with rupture as the initial clinical presentation, resulting in significant morbidity and mortality. Consequential intracranial hemorrhage and increased intracranial hypertension can result in worsening hemodynamic stability and neurologic sequelae. While standard treatments of pAVFs include endovascular embolization, microsurgical disconnection, or a hybrid approach, patients who present with significantly increased ICP refractory to medical management can potentially benefit from immediate decompressive craniectomy even in the absence of cerebral edema or lateralizing pathology, as seen in our case. This approach may prove effective in acute stabilization of intracranial pressure secondary to intraventricular hemorrhage due to ruptured pAVF and allows for a good neurologic outcome; however, it warrants further study to better understand the role of this therapy in various disease mechanisms.

## References

[1] Alurkar A, Karanam LS, Nayak S, Ghanta RK (2016). Intracranial pial arteriovenous fistulae: diagnosis and treatment techniques in pediatric patients with review of literature. J Clin Imaging Sci.

[2] Etter MM, Guzman R, Psychogios MN, Soleman J (2025). Pial arteriovenous fistula with a large intraparenchymal hemorrhage in a 9-year-old child: a case report and case-based mini review. Childs Nerv Syst.

[3] Li J, Ji Z, Yu J (2023). Angioarchitecture and prognosis of pediatric intracranial pial arteriovenous fistula. Stroke Vasc Neurol.

[4] Thrash GW, Hale AT, Feldman MJ (2024). Pediatric non-galenic pial arteriovenous fistula's characteristics and outcomes: a systematic review. Childs Nerv Syst.

[5] Lim J, Kuo CC, Waqas M (2023). A systematic review of non-galenic pial arteriovenous fistulas. World Neurosurg.

[6] Requejo F, Jaimovich R, Marelli J, Zuccaro G (2015). Intracranial pial fistulas in pediatric population. Clinical features and treatment modalities. Childs Nerv Syst.

[7] LoPresti MA, Goethe EA, Lam S (2020). Surgical strategies for management of pediatric arteriovenous malformation rupture: the role of initial decompressive craniectomy. Childs Nerv Syst.

[8] Srinivasan VM, Gressot LV, Daniels BS, Jones JY, Jea A, Lam S (2016). Management of intracerebral hemorrhage in pediatric neurosurgery. Surg Neurol Int.

[9] Stricker S, Boulouis G, Benichi S (2021). Acute surgical management of children with ruptured brain arteriovenous malformation. J Neurosurg Pediatr.

[10] Stricker S, Boulouis G, Benichi S (2020). Hydrocephalus in children with ruptured cerebral arteriovenous malformation. J Neurosurg Pediatr.

[11] Konar SK, Dinesh YS, Shukla D (2024). Decompressive craniectomy in children: indications and outcome from a tertiary centre. Childs Nerv Syst.

[12] Ahn JH, Phi JH, Kang HS (2010). A ruptured middle cerebral artery aneurysm in a 13-month-old boy with Kawasaki disease. J Neurosurg Pediatr.

[13] Meyer PG, Orliaguet GA, Zerah M (2000). Emergency management of deeply comatose children with acute rupture of cerebral arteriovenous malformations. Can J Anaesth.

[14] Ranger A, Szymczak A, Fraser D, Salvadori M, Jardine L (2009). Bilateral decompressive craniectomy for refractory intracranial hypertension in a child with severe ITP-related intracerebral haemorrhage. Pediatr Neurosurg.

